# Association Between Mental Health Literacy and Its Dimensions with Adolescent Depression and Anxiety: A Cross-Sectional Study Among 5759 Adolescents in China

**DOI:** 10.3390/bs16061027

**Published:** 2026-06-18

**Authors:** Zhihan Jiang, Xing Wang, Yuteng Luo, Zeyun Hu, Shibin Wang, Yanbin Liu, Heng Wu

**Affiliations:** 1 Department of Psychosomatic Medicine, Shanghai Tongji Hospital, Tongji University School of Medicine, Shanghai 200065, China; 2432599@tongji.edu.cn (Z.J.);; 2 Department of Neurosis and Psychosomatic Diseases, Huzhou Third Municipal Hospital, The Affiliated Hospital of Huzhou University, Huzhou 313000, China; 3 School of Clinical Medicine, Zhejiang Chinese Medical University, Hangzhou 310053, China; 4 Guangdong Mental Health Center, Guangdong Provincial People’s Hospital (Guangdong Academy of Medical Sciences), Southern Medical University, Guangzhou 510080, China; 5 National Center for Mental Health, Beijing 100027, China

**Keywords:** adolescent, mental health literacy, depression, anxiety, machine learning, China

## Abstract

Introduction: Adolescent depression and anxiety are major public health concerns. Previous studies showed that low mental health literacy is associated with depressive and anxiety symptoms. However, how its core dimensions—knowledge, attitudes, and skills—differentially relate to emotional symptoms remains unclear. Methods: A school-based survey was conducted among 6400 adolescents in Guangdong, China. Eligible participants completed the MHL questionnaire and assessments for depressive and anxiety symptoms. We assessed whether MHL was associated with depressive and anxiety symptoms in adolescents. Machine learning algorithms with SHAP analysis were applied to explore complex associations and validate key findings. Results: A total of 5759 adolescents were included. MHL and the knowledge dimension were negatively associated with depressive and anxiety symptoms. The attitudes dimension showed a negative association with both mental health outcomes (depression: OR = 0.83; anxiety: OR = 0.84) and machine learning confirmed attitudes as the key factor. Skills were unrelated to depressive symptoms. At the highest quartile, skills showed a positive association with anxiety symptoms (OR = 1.29). Conclusions: The attitudes dimension is negatively associated with adolescent depressive and anxiety symptoms and emerged as a key feature in ML identification models.

## 1. Introduction

Depression and anxiety are major contributors to disease burden and functional impairment in adolescents worldwide (Castaldelli-Maia et al., 2025). They are linked to higher risks of substance abuse, self-injury, suicide, and poor physical health (Clayborne et al., 2019; Essau et al., 2014). An estimated 10% to 20% of adolescents experience depressive episodes or anxiety disorders (including generalized anxiety disorder, separation anxiety, and panic disorder) (Scafe et al., 2025; Shorey et al., 2022). They are not only highly comorbid but also often follow a chronic and recurrent course (Pollard et al., 2023; Rybak et al., 2021). Although pharmacological and psychological treatments are evidence-based, medication may cause adverse effects and poor adherence (Scafe et al., 2025). Effective psychological therapies often require long treatment cycles and have limited accessibility (Bodden et al., 2008).

Mental health literacy (MHL) has gained increasing attention from researchers and public health policymakers due to its modifiable and feasible nature among various mental health promotion initiatives (Asplund et al., 2025; Shorey et al., 2022). Jorm et al. originally defined MHL as “knowledge and beliefs about mental disorders which aid their recognition, management, or prevention (Jorm et al., 1997).” With theoretical and practical advancements, this concept has evolved to include the integrated application of knowledge (basic mental health principles, knowledge of disorders and treatment, mind–body health), attitudes (toward prevention, help-seeking, treatment, and stigma), and skills (recognizing disorders and providing mental health first aid) (Kutcher et al., 2016). Research shows that high MHL facilitates early recognition of mental disorders, reduces stigma, and promotes positive attitudes toward seeking help (Asplund et al., 2025). Conversely, low MHL is associated with recent depression and anxiety, suggesting a key role in preventing emotional disorders (Zhong et al., 2024). Unfortunately, low MHL may be common across all age groups, particularly among adolescents in low- and middle-income countries (Lam, 2014; Renwick et al., 2024). It is crucial to examine the specific associations between its distinct dimensions (knowledge, attitudes, skills) and adolescent emotional symptoms (Bjørnsen et al., 2019). This further enhances our understanding of the relevant content.

Traditional statistical methods (e.g., logistic regression) may have limitations in examining the complex, potentially non-linear, or interactive relationships between multidimensional MHL and emotional symptoms (Rajula et al., 2020). Machine learning (ML) offers the ability to capture non-linear relationships and interactions (Geurgas et al., 2025). Combined with the Shapley Additive Interpretation (SHAP) framework, it allows quantification and visualization of each variable’s specific contribution to model identification (Chu et al., 2025).

This study aims to use ML as a complementary approach to regression, to examine complex relationships between MHL dimensions and adolescent depressive and anxiety symptoms, and to validate key feature importance.

## 2. Methods

### 2.1. Sample and Procedures

This survey used a large school-based sample of adolescents in China. The study population consisted of adolescent students from secondary education schools in Guangdong Province, one of China’s most populous and socio-economically diverse provinces. Participants were recruited through a school-based centralized survey conducted between September and December 2023. The sample was drawn from multiple secondary schools across different urban and rural districts of the province, covering all grade levels from 7 to 12. The study aimed to assess MHL, service needs, and psychological well-being among adolescents. We used electronic questionnaires that required respondents to complete all fields before submission to ensure that all responses were complete. Before the survey was conducted, the planned sample size was 6400 people. Some selected participants were unable to participate. Finally, 5759 participants provided valid data, with a response rate of 90.0%.

The study was carried out following the principles of the Declaration of Helsinki. The Ethics Review Committee of Guangdong Provincial People’s Hospital approved the study (approval number: KY-Z-2022-063-02). All participants and their guardians gave informed consent prior to the survey.

### 2.2. Measurements

#### 2.2.1. Mental Health Literacy

Adolescent MHL was assessed using China’s National Mental Health Literacy Questionnaire (MHLQ), a standardized instrument developed by the Institute of Psychology, Chinese Academy of Sciences (Zhong et al., 2024). This standardized instrument evaluates three core dimensions: knowledge (20 true or false items; score range 0–100), attitudes (8 items on a 4-point Likert scale; score range 8–32), and skills (16 items; assessed via case vignettes; score range 0–40). A total MHL score is derived from the sum. Higher scores indicate better mental health literacy in each dimension. The scale demonstrated good reliability in this study (Total Cronbach’s α = 0.804; subscale α: 0.80–0.85).

#### 2.2.2. Depression

Adolescent depressive symptoms were assessed using the 9-item short Chinese version of the Center for Epidemiologic Studies Depression Scale (CES-D) (He et al., 2013). Respondents rate the frequency of symptoms over the past week on a 4-point scale (0–3), yielding a total score ranging from 0 to 27. The scale has a two-factor structure (negative and positive affect) and demonstrates good validity, correlating highly with the original 20-item CES-D (r = 0.94–0.96). Radloff determined that a score between 16 and 28 indicated a tendency towards depression (20% of the population) and a high risk of depression (5% of the population), respectively, in the original CES-D (Radloff, 1977). In this study, a score of ≥17 was used to indicate a high risk of depression. This is consistent with the survey conducted in the Chinese population (He et al., 2013). The internal consistency in this sample was acceptable (Cronbach’s α = 0.70).

#### 2.2.3. Anxiety

Adolescent anxiety symptoms were assessed using the Chinese version of the Generalized Anxiety Disorder Scale-7 (GAD-7) (Tong et al., 2016). Respondents rate the frequency of seven core anxiety symptoms over the past two weeks on a 4-point scale (0 = not at all to 3 = nearly every day). The total score ranges from 0 to 21. For this study, a total score of ≥10 was used to define high risk for current anxiety (yes/no). The Cronbach’s α in this study was 0.92.

### 2.3. Confounders

We assessed sociodemographic and health-related information, including age, gender, grade, residence, only child (OC), boarding, family economic situation (FES), exercise, eating habit (EH), spend on electronic screen devices (SOESD), current myopia status (CMS), BMI, Parental parenting style (PPS), and parental relationship (PR). Age was categorized into four groups: early adolescence (≤13 years), middle adolescence (14–15 years), late-middle adolescence (16–17 years), and late adolescence (≥18 years). Residence was classified as rural or urban. FES was measured using a five-item ordinal scale ranging from <¥3500/month to >¥20,000/month and was categorized as poor (<¥3500), below average (¥3500–¥7999), average (¥8000–¥11,999), above average (¥12,000–¥19,999), and affluent (≥¥20,000). Exercise was classified into five categories: never/rarely, 1–3 times/month, 1–2 times/week, 3–5 times/week, and daily/almost daily. Eating habit was categorized as regular three meals daily, regular two meals daily, regular multiple meals daily (more than three meals), or irregular. SOESD was divided into five categories: <2 h, 2–4 h, 4–6 h, 6–8 h, and >8 h. CMS was classified into five categories: no myopia, mild myopia (<100 diopters), low-moderate myopia (100–299 diopters), high-moderate myopia (300–599 diopters), and high myopia (≥600 diopters). BMI was categorized according to WHO standards as underweight (BMI < 18.5), normal weight (BMI 18.5–24.9), overweight (BMI 25–29.9), and obese (BMI ≥ 30). PPS was assessed using a single-item measure and classified into five categories: strict mother/kind father, strict father/kind mother, kind parents, strict parents, and not applicable. PR was assessed using a single-item measure and classified into five categories: very harmonious, relatively harmonious, average, relatively discordant, and very discordant.

### 2.4. Statistical Analysis

The initial analysis of the dataset involved descriptive statistics. Baseline characteristics were compared between adolescents with and without depressive and anxiety symptoms. Continuous variables were presented as means ± standard deviations, and categorical variables as frequencies (percentages). For continuous variables, independent sample *t*-tests or analysis of variance (ANOVA) were used for normally distributed data, while the Mann–Whitney U test was applied for non-normally distributed data. Categorical variables were analyzed using Pearson’s chi-square test or Fisher’s exact test. Multivariable logistic regression was used to examine associations between MHL (including its three domains: knowledge, attitudes, and skills) and adolescent depression or anxiety. To reduce confounding, we constructed three sequential models: Model 1—unadjusted; Model 2—adjusted for gender and age; Model 3—further adjusted for residence, OC, boarding, grade, BMI, exercise, EH, SOESD, CMS, FES, PPS, and PR. Tests for trends were computed using quartiles of MHL and its dimensions. We used restricted cubic spline (RCS) regression with three knots to explore potential non-linear relationships between MHL (and its dimensions) and depression or anxiety in adolescents. All statistical analyses were performed using Python (version 3.14.3) and R (version 4.3.0). Specifically, R was used for data management, group comparisons, multivariable logistic regression, RCS analysis, and feature selection. Python was used for all ML model development, evaluation, visualization, and interpretability analysis. All reported P values were two-sided, and statistical significance was set at *p* ≦ 0.05.

### 2.5. Feature Selection and Machine Learning Identification Model Construction

In this study, we developed ML models to examine whether MHL and its dimensions can identify adolescent depressive and anxiety symptoms. The full dataset (N = 5759) was randomly split into a training set (70%) and a test set (30%) with a random seed of 100. The test set was kept independent and was used only for final evaluation. In the training set only, we used two complementary methods to select the discriminative factors from sociodemographic characteristics, lifestyle behaviors, and MHL dimensions: Boruta (a random forest-based wrapper method) and LASSO regression (adds a penalty function and shrinks coefficients). Importantly, only the three MHL sub-dimensions (knowledge, attitudes, skills) were entered as candidate features, the total MHL score was excluded to avoid potential part–whole dependency and identity redundancies. Features selected by both methods were retained for subsequent model building. Boruta and LASSO were implemented using the Boruta (version 8.0.0) and glmnet packages in R.

Based on the selected features, we built classification models using ten classic ML algorithms: logistic regression (LR), random forest (RF), Gradient Boosting (GBDT), eXtreme Gradient Boosting (XGBoost), Light Gradient Boosting Machine (LightGBM), K-Nearest Neighbors (KNNs), Decision Tree (DT), Extra Tree (ET), Adaptive Boosting (AdaBoost) and Naive Bayes (NB). We applied the Synthetic Minority Over-sampling Technique (SMOTE) with a sampling strategy of 0.5 to address class imbalance in the training set. To prevent information leakage, both feature scaling (StandardScaler) and SMOTE were embedded inside an imblearn.pipeline.Pipeline. This ensured that within each cross-validation (CV) fold, the scaler was fitted only to the training sub-fold, and SMOTE generated synthetic samples exclusively from that same training sub-fold, leaving the validation sub-fold untouched. For each of the ten ML models, we used Bayesian optimization (BayesSearchCV) with 30 iterations to search the hyperparameter space. The search was guided by 10-fold cross-validation on the training set. The pipeline containing scaling, SMOTE, and the classifier was passed to BayesSearchCV, so that within each CV fold, all preprocessing steps were correctly reapplied. The best hyperparameters were selected based on the average cross-validated area under the ROC curve (AUC). After obtaining the optimal hyperparameters for each algorithm, we retrained the model on the entire training set (using the same pipeline) and evaluated its performance on the independent test set. Performance metrics included AUC, accuracy, precision, recall, F1 score, and specificity. To interpret the best-performing model (selected based on the test AUC and overall balance of metrics), we used SHAP to visualize feature importance and the contribution to each feature.

## 3. Results

The final analysis included 5759 valid responses (46.9% male, 53.1% female). Participants ranged in age from 10 to 20 years and covered all grades from junior high school (grades 7 to 9) to high school or equivalent vocational education (grades 10 to 12). The sample was drawn from both urban (77.0%) and rural (23.0%) areas, reflecting the socioeconomic diversity of the region.

### 3.1. Baseline Characteristic

This study included 5759 adolescents. They were divided into two groups: those with depressive symptoms (*n* = 516) and those without (*n* = 5243), and those with anxiety symptoms (*n* = 746) and those without (*n* = 5013). More than half of the overall sample were female (53.1%, *n* = 3056). The proportion of females was higher in both the depression group (68.4%, *n* = 353) and the anxiety group (64.2%, *n* = 479). Compared to their counterparts, adolescents with depression and anxiety tended to be older, engage in less physical activity, have irregular eating habits, spend more time on screens, have more severe myopia, come from poorer families, experience stricter parenting, and live in less harmonious family environments. The main demographic and behavioral characteristics of each group are presented in Table 1 and Table 2. The remaining parts are contained in the Supplementary Materials (Tables S1 and S2).

### 3.2. Association Between Adolescent MHL and Depression Risk

Multivariable logistic regression showed that after adjusting for confounders, MHL as a continuous variable was negatively associated with depression risk in adolescents (OR = 0.98, 95% CI: 0.979–0.988, *p* < 0.001). This association remained significant across all quartiles, with a dose–response trend (*p* for trend < 0.001). In Model 3, adolescents with high MHL (Q4) had significantly lower depression risk than those with low MHL (Q1), with approximately 68% lower risk (OR = 0.32, 95% CI: 0.23–0.43, *p* < 0.001). Similarly, knowledge and attitudes as continuous variables were each negatively associated with depression risk (knowledge: OR = 0.98, 95% CI: 0.980–0.990, *p* < 0.001; attitudes: OR = 0.83, 95% CI: 0.811–0.856, *p* < 0.001). These associations remained significant across all quartiles, with dose–response trends (*p* for trend < 0.001 for both). Compared to their low-level counterparts (Q1), adolescents with high knowledge (Q4) had 55% lower depression risk (OR = 0.45, 95% CI: 0.34–0.60, *p* < 0.001), and those with high attitudes (Q4) had 81% lower risk (OR = 0.19, 95% CI: 0.12–0.28, *p* < 0.001). In contrast, skills showed no significant association with depression risk in any model, either as a continuous variable (OR = 1.01, 95% CI: 0.989–1.033, *p* = 0.34) or across quartiles (*p* for trend = 0.36) (Table 3).

### 3.3. Association Between Adolescent MHL Level and Anxiety Risk

Multivariable logistic regression showed that after adjusting for confounders, MHL as a continuous variable was negatively associated with anxiety risk in adolescents (OR = 0.98, 95% CI: 0.982–0.990, *p* < 0.001). This association remained significant across all quartiles, with a dose–response trend (*p* for trend < 0.001). In Model 3, adolescents with high MHL (Q4) had significantly lower anxiety risk than those with low MHL (Q1), with approximately 59% lower risk (OR = 0.41, 95% CI: 0.32–0.53, *p* < 0.001). Similarly, knowledge and attitudes as continuous variables were each negatively associated with anxiety risk (knowledge: OR = 0.99, 95% CI: 0.983–0.992, *p* < 0.001; attitudes: OR = 0.84, 95% CI: 0.816–0.856, *p* < 0.001). These associations remained significant across all quartiles, with dose–response trends (*p* for trend < 0.001 for both). Compared to their low-level counterparts (Q1), adolescents with high knowledge (Q4) had a 36% lower anxiety risk (OR = 0.64, 95% CI: 0.50–0.80, *p* < 0.001), and those with high attitudes (Q4) had an 83% lower risk (OR = 0.17, 95% CI: 0.12–0.24, *p* < 0.001). In contrast, skills showed mixed associations with anxiety risk. After adjusting for confounders, skills as a continuous variable was positively associated with anxiety risk (OR = 1.02, 95% CI: 1.000–1.038, *p* = 0.049). Across the three models, skills was significantly associated with anxiety only at the highest quartile (Q4), with a dose–response trend (*p* for trend < 0.05). In the fully adjusted Model 3, adolescents with high skills (Q4) had significantly higher anxiety risk than those with low skills (Q1) (OR = 1.29, 95% CI: 1.02–1.62, *p* = 0.031) (Table 4).

### 3.4. RCS Analysis

**Figure 1:**
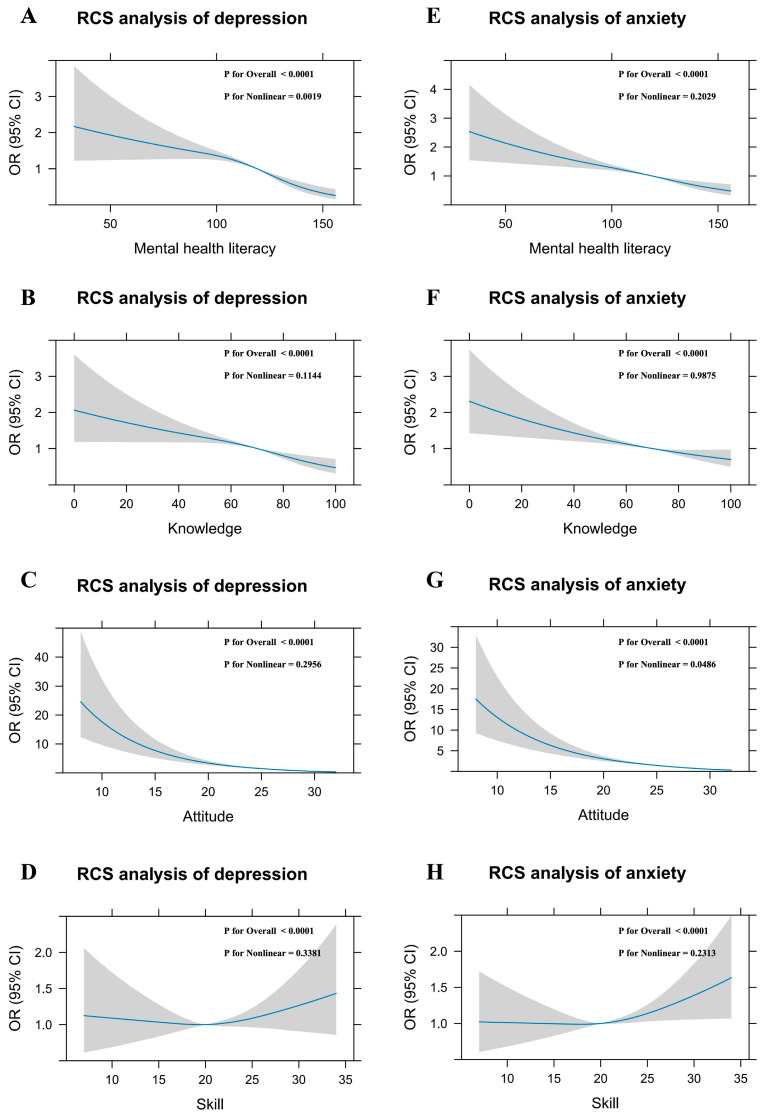
(**A**–**H**): RCS analysis. Adjusted for gender, age, residence, OC, boarding, grade, BMI, exercise, EH, SOESD, CMS, FES, PPS, and PR.

We performed RCS analyses in the fully adjusted logistic regression model (Model 3). The results showed that MHL (including knowledge, attitudes, and skills) was significantly associated with the risk of depression and anxiety in adolescents (*p* for overall < 0.0001) (Figure 1A–H). For depression, a significant non-linear effect was detected for MHL (*p* for non-linear = 0.0019), indicating that the dose–response relationship between MHL and depression followed a non-linear pattern (Figure 1A). In contrast, no significant non-linear effects were found for knowledge, attitudes, or skills (*p* for non-linear = 0.1144, 0.2956, and 0.3381, respectively), suggesting that their dose–response relationships with depression followed linear patterns (Figure 1B–D). For anxiety, a significant non-linear effect was detected for attitudes (*p* for non-linear = 0.0486), indicating that the dose–response relationship between attitudes and anxiety followed a non-linear pattern (Figure 1G). No significant non-linear effects were found for MHL, knowledge, or skills (*p* for non-linear = 0.2029, 0.9875, and 0.2313, respectively), suggesting that their dose–response relationships with anxiety followed linear patterns (Figure 1E,F,H).

### 3.5. Feature Selection and Model Performance

We used the Boruta algorithm, a random forest-based method, to identify reliable variables for detecting depression and anxiety risk in adolescents. For depression, the top five variables ranked by importance were attitudes, PR, grade, age, and SOESD. For anxiety, the top five were attitudes, EH, PR, SOESD, and exercise.

**Figure 2:**
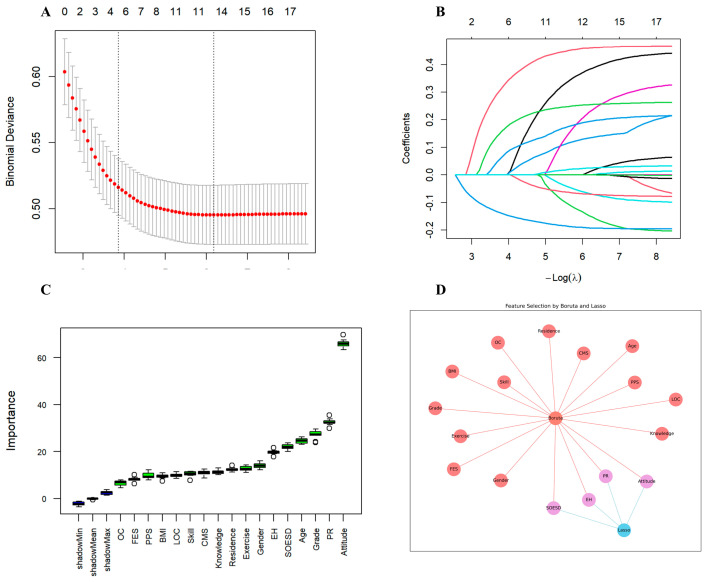
Factor screening results of depression risk. (**A**) Factor screening based on the LASSO regression model; (**B**) LASSO regression model screening variable trajectories; (**C**) Boruta; (**D**) common factors between Boruta and LASSO.

**Figure 3:**
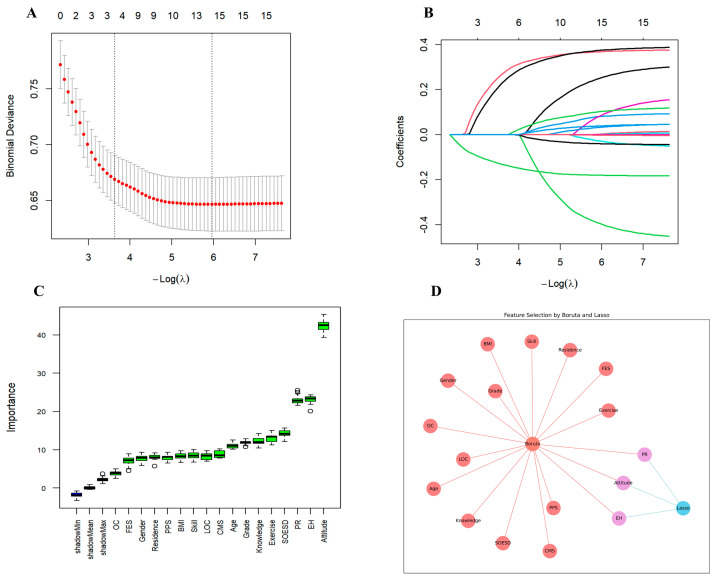
Factor screening results of anxiety risk. (**A**) Factor screening based on the LASSO regression model; (**B**) LASSO regression model screening variable trajectories; (**C**) Boruta; (**D**) common factors between Boruta and LASSO.

We also applied LASSO regression, which selects variables by adding a penalty function to the model. For depression, LASSO identified attitudes, PR, EH, and SOESD. For anxiety, it identified attitudes, PR, and EH. By comparing the results from Boruta and LASSO, we derived a subset of features selected by both methods. These features were then used to build identification models (Figure 2 and Figure 3).

As a sensitivity check, we also compared the model that included the total MHL score with its sub-dimensions. The attitudes dimension remained the top-ranked feature in SHAP analysis, and the metrics of models were stable.

**Figure 4:**
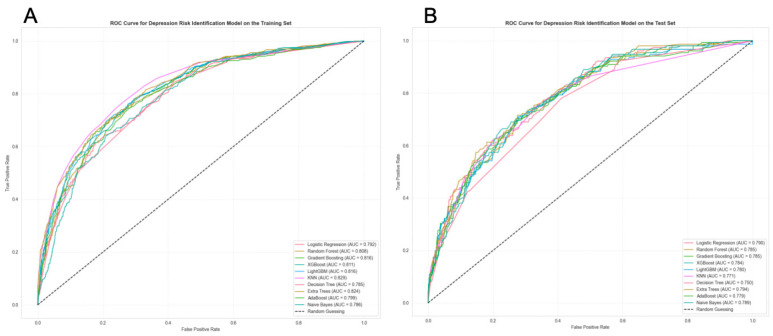
(**A**,**B**): ROC graphs of 10 types of machine learning in the training set and test set of depression.

We developed ten ML models to explore complex patterns and validate feature importance for depressive and anxiety symptoms. For depression, the models with the highest AUC in the training set were KNN (AUC = 0.829), ET (AUC = 0.824), Gradient Boosting (AUC = 0.816), LightGBM (AUC = 0.816), XGBoost (AUC = 0.811), RF (AUC = 0.808), and AdaBoost (AUC = 0.799). In the test set, the top performers were ET (AUC = 0.794), LR (AUC = 0.790), NB (AUC = 0.789), Gradient Boosting (AUC = 0.785), and RF (AUC = 0.785) (Figure 4). We calculated and compared the accuracy, sensitivity, specificity, positive predictive value (PPV), negative predictive value (NPV), and F1 score for each model (Table S3). Calibration plots have also been added (Figure S1A). Based on these metrics, LR was selected as the optimal model.

**Figure 5:**
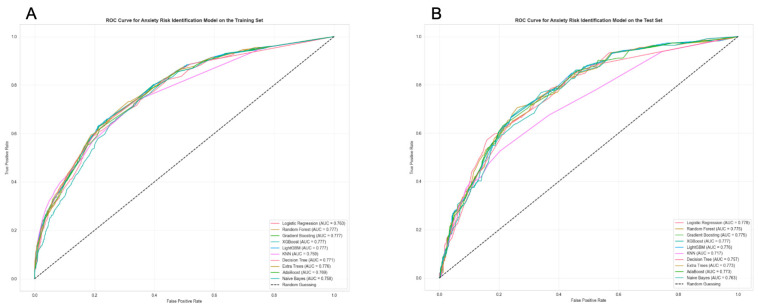
(**A**,**B**): ROC graphs of 10 types of machine learning in the traning set and test of anxiety.

For anxiety, the models with the highest AUC in the training set were RF (AUC = 0.777), Gradient Boosting (AUC = 0.777), and LightGBM (AUC = 0.777). In the test set, the top performers were LR (AUC = 0.778), XGBoost (AUC = 0.777) and LightGBM (AUC = 0.776) (Figure 5). We calculated and compared the accuracy, sensitivity, specificity, PPV, NPV, and F1 score for each model (Table S4). Calibration plots have also been added (Figure S1B). LR was ultimately selected as the optimal model.

### 3.6. Model Interpretability Analysis Based on SHAP

**Figure 6:**
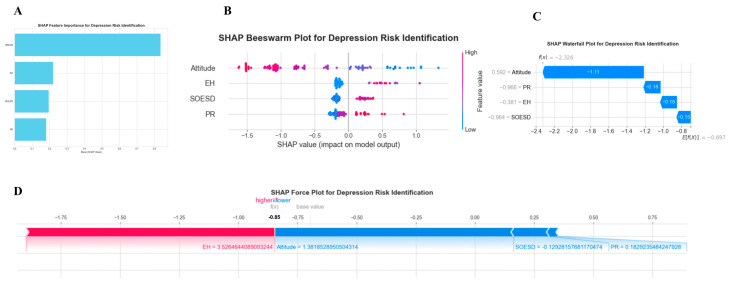
Interpretability analysis for depression risk of LR models. (**A**): Importance ranking plot of features. (**B**): SHAP beeswarm plot. (**C**,**D**): Interpretability analysis of 2 independent samples.

**Figure 7:**
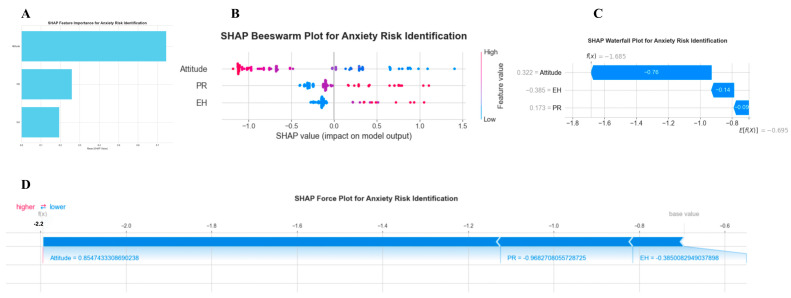
Interpretability analysis for depression risk of LR models. (**A**): Importance ranking plot of features. (**B**): SHAP beesarm plot. (**C**,**D**): Interpretability analysis of 2 independent samples.

We assessed the relative importance of multiple factors influencing adolescent depression and anxiety. Figure 6A shows the feature importance ranking for identifying depression risk, and Figure 7A shows the ranking for anxiety risk. Figure 6B and Figure 7B provide visual representations of these rankings. Each dot represents an individual sample, with colors ranging from blue to red, indicating low to high feature values. The vertical axis displays the feature importance ranking, and the color gradient reflects the relationship and distribution between each feature value and its SHAP value. To better understand the model’s decision-making process at the individual level, we performed detailed interpretability analysis on two representative samples, as shown in Figure 6C,D and Figure 7C,D. By visualizing the SHAP values for these samples, we can determine how each feature influenced the model’s identification for these specific cases.

## 4. Discussion

This cross-sectional study of 5759 Chinese adolescents examined associations between MHL dimensions and depressive/anxiety symptoms. Key findings include higher MHL and knowledge correlated with fewer emotional symptoms, the attitudes dimension is inversely associated with adolescent depressive and anxiety symptoms and emerged as a key feature in ML identification models. The skill dimension was unrelated to depressive symptoms and positively linked to anxiety symptoms at high levels (Q4).

Several findings highlight the importance of early screening and identification for adolescents with emotional problems (Bista et al., 2025). In our sample of Chinese adolescents, the prevalence of elevated risk of depression and anxiety was 8.96% (516/5759) and 12.95% (746/5759), respectively. This is likely associated with multiple challenges faced by adolescents in this age group within China’s specific sociocultural context, including intense academic competition, high parental educational expectations, and excessive internet use (Chen et al., 2023; Yu et al., 2023). Consistent with existing literature, we found that females were more likely to suffer from depressive and anxiety symptoms, suggesting that females may be a more vulnerable subgroup during adolescence (Afifi, 2007; Piccinelli & Wilkinson, 2000). Adolescents with unhealthy lifestyle factors—including irregular eating habits, less physical activity, and longer daily screen time—had higher risk of depression and anxiety. Family environment also mattered. We observed a higher risk of depression and anxiety among adolescents from families with poorer economic status (below average), discordant parental relationships, and harsh parenting styles (particularly when both parents were harsh). Previous research suggests that economic stress may limit adolescents’ access to family resources, frequent family conflict may erode adolescents’ sense of safety and belonging, and parenting styles lacking warmth or exhibiting excessive control may impair the development of emotion regulation capacities (Westrupp et al., 2023; Yang et al., 2024).

Our results showed that MHL was negatively associated with recent depressive and anxiety symptoms among Chinese adolescents. This aligns with previous cross-sectional studies conducted among Chinese adults and older populations (Ding et al., 2022; Zhong et al., 2024). These studies indicated that the MHL may be associated with mental health across different age groups, even though specific mental health concerns (e.g., academic anxiety in adolescents, workplace stress in adults, loneliness in older adults) and available help-seeking resources evolve across developmental stages (Bhui et al., 2016; da Silva, 2024; Lee et al., 2024).

This study further revealed the associations between different MHL dimensions and adolescent depressive and anxiety symptoms. The attitudes dimension showed a negative association with depressive and anxiety symptoms and emerged as a key feature in ML identification models. Positive attitudes—including non-stigmatizing views of mental illness, lower personal stigma, and willingness to seek help—may be key factors facilitating timely help-seeking and reducing psychological distress (Kreiner, 2025). Previous findings from a stigma reduction program (End the Silence) among US high school students support this, with the intervention group showing significant advantages in help-seeking, stereotype reduction, and improved negative emotions (Bruckner Tim et al., 2012). We found that the knowledge dimension was negatively associated with adolescent depressive and anxiety symptoms. However, compared to attitudes, a significant reduction in depression risk was only observed at the highest knowledge level (Q4). For anxiety, no clear risk reduction was observed across middle-to-high knowledge levels (Q3–Q4). This suggests that knowledge may serve as a foundation for behavior (Guo et al., 2020). Knowledge likely increases adolescents’ understanding of mental health and illness, thereby reducing stigma and promoting help-seeking behaviors (Yin et al., 2020). Previous research has suggested that knowledge may underpin the formation of appropriate attitudes (Wei et al., 2015). A meta-analysis indicated that gains in mental health knowledge following interventions tended to diminish over time, as knowledge may be forgotten (Amado-Rodríguez et al., 2022). This may partially explain the limited effectiveness of some interventions focused primarily on knowledge dissemination (Ma et al., 2023). We found that the skills dimension was unrelated to depressive symptoms and, unexpectedly, was positively associated with anxiety symptoms at the highest skill level (Q4). Given the cross-sectional design, this study cannot determine whether higher skills increase anxiety risk, whether adolescents with anxiety symptoms develop better recognition skills, or whether other factors (e.g., residual confounding, limitation of the skill subscale based on vignette assessment, and cultural bias) explain both higher skills and anxiety risk. One possible explanation is that within China’s high-pressure educational environment, adolescents with higher skill levels may be more sensitive to recognizing emotional and behavioral disturbances in peers (Jiang et al., 2021). However, without adequate capacity for effective response and support, this heightened sensitivity might temporarily increase their own anxiety symptoms. A previous adolescent mental health first aid (MHFA) program in Australia showed that trained adolescents significantly increased supportive first aid intentions and reduced stigmatizing beliefs (Morgan et al., 2020). This suggests that the value of the skills dimension may depend on whether it completes the full cycle from recognition to action. MHFA programs redefine the role boundary for adolescents—effective help is not about “solving problems alone” but rather helping distressed peers seek professional support (Liang et al., 2023). This role repositioning alleviates burden, transforming skills from a source of responsibility into a tool for support. In China, school-based mental health services are relatively scarce, and societal stigma about psychological problems persists, potentially hindering adolescents from actively seeking help (Qu et al., 2024). A meta-analysis of MHFA studies indicated that MHFA improved mental health knowledge, reduced negative attitudes, and enhanced supportive behaviors (Morgan et al., 2018). Nevertheless, most samples were from high-income countries (e.g., UK, USA, Australia), suggesting that conclusions should be cautiously extrapolated to low- and middle-income countries and culturally diverse populations. Intervention effects on the skills dimension may vary due to differences in cultural beliefs, healthcare resources, and help-seeking pathways (Morgan et al., 2019).

Our study has several implications. Theoretically, this study advances the literature by moving beyond treating MHL as a single construct and separately examining how its distinct dimensions relate to adolescent depressive and anxiety risk. Methodologically, applying interpretable machine learning provides a transparent framework for analyzing complex multivariate relationships in mental health research, demonstrating the potential of this approach for future studies.

Several limitations should be considered when interpreting our results. First, this was a cross-sectional study, which precludes causal inferences about relationships between MHL dimensions and adolescent emotional disorders. Accordingly, in our machine learning models, we specifically use the term “identify” rather than “predict” to avoid implying causality. Second, measures were self-reported, which are subject to social desirability and recall biases. Adolescents may underreport depressive or anxiety symptoms due to stigma, or overreport MHL-related attitudes to appear more socially aware. Although the anonymous electronic survey reduced these biases and the recall periods were short (one week for CES-D, two weeks for GAD-7), they cannot be eliminated. Third, the absence of sampling weights also precludes generalization to the entire adolescent population of Guangdong. Additionally, single-item measures were used to assess parenting styles and family atmosphere. While this approach reduces respondent burden in large-scale surveys, it may inadequately capture complex behavioral patterns and introduce measurement bias. Fourth, the ML models were trained and validated on a single cross-sectional sample of Chinese adolescents. Their generalizability to other age groups and cultural contexts needs to be cautioned. Although SHAP analysis helped quantify the relative importance of MHL dimensions, it cannot clarify causal pathways or directional relationships between MHL and depressive or anxiety symptoms. Future research should be conducted longitudinally among people from different cultural backgrounds and age groups. Fifth, although we employed stepwise regression, variables including demographic, family, and lifestyle factors may introduce over-adjustment bias. Some variables (e.g., parenting style, family relationship, exercise, screen time) may act as mediators rather than confounders, potentially attenuating true associations. The cross-sectional design cannot distinguish confounding from mediation, which should be clarified in future longitudinal studies.

## 5. Conclusions

The attitudes dimension is negatively associated with adolescent depressive and anxiety symptoms and emerged as a key feature in ML identification models.

## Figures and Tables

**Table 1:** Characteristics of participants by depressive symptom status.

	All	No Depressive Symptoms	Depressive Symptoms	*p*. Overall
	*N = 5759*	*N = 5243*	*N = 516*	
Age:				<0.001
13_and_below	1532 (26.60%)	1444 (27.54%)	88 (17.05%)	
14_15	2137 (37.11%)	1930 (36.81%)	207 (40.12%)	
16_17	1843 (32.00%)	1647 (31.41%)	196 (37.98%)	
18_and_above	247 (4.29%)	222 (4.23%)	25 (4.84%)	
Gender:				<0.001
male	2703 (46.94%)	2540 (48.45%)	163 (31.59%)	
female	3056 (53.06%)	2703 (51.55%)	353 (68.41%)	
Residence:				0.358
Urban	4432 (76.96%)	4026 (76.79%)	406 (78.68%)	
Rural	1327 (23.04%)	1217 (23.21%)	110 (21.32%)	
Exercise:				<0.001
Never/Rarely	532 (9.24%)	431 (8.22%)	101 (19.57%)	
1–3 times/month	1318 (22.89%)	1169 (22.30%)	149 (28.88%)	
1–2 times/week	1831 (31.79%)	1699 (32.41%)	132 (25.58%)	
3–5 times/week	910 (15.80%)	848 (16.17%)	62 (12.02%)	
Daily or almost daily	1168 (20.28%)	1096 (20.90%)	72 (13.95%)	
Eating habit:				<0.001
Regular three meals a day	4886 (84.84%)	4541 (86.61%)	345 (66.86%)	
Regular two meals a day	319 (5.54%)	267 (5.09%)	52 (10.08%)	
Regular multiple meals a day (more than three)	281 (4.88%)	242 (4.62%)	39 (7.56%)	
Irregular eating pattern	273 (4.74%)	193 (3.68%)	80 (15.50%)	
Spend on electronic screen devices:				<0.001
Less than 2 h	3860 (67.03%)	3621 (69.06%)	239 (46.32%)	
2 to less than 4 h	1156 (20.07%)	1008 (19.23%)	148 (28.68%)	
4 to less than 6 h	458 (7.95%)	396 (7.55%)	62 (12.02%)	
6 to less than 8 h	164 (2.85%)	134 (2.56%)	30 (5.81%)	
8 h or more	121 (2.10%)	84 (1.60%)	37 (7.17%)	
Parental parenting style:				<0.001
Strict father, kind mother	1224 (21.25%)	1123 (21.42%)	101 (19.57%)	
Kind father, strict mother	979 (17.00%)	888 (16.94%)	91 (17.64%)	
Strict father, strict mother	609 (10.57%)	519 (9.90%)	90 (17.44%)	
Kind father, kind mother	2292 (39.80%)	2155 (41.10%)	137 (26.55%)	
Not applicable	655 (11.37%)	558 (10.64%)	97 (18.80%)	
Parental Relationship:				<0.001
Very harmonious	2309 (40.09%)	2203 (42.02%)	106 (20.54%)	
Relatively harmonious	2401 (41.69%)	2211 (42.17%)	190 (36.82%)	
Average	787 (13.67%)	650 (12.40%)	137 (26.55%)	
Not very harmonious	180 (3.13%)	117 (2.23%)	63 (12.21%)	
Very disharmonious	82 (1.42%)	62 (1.18%)	20 (3.88%)	
Knowledge	68.39 (18.33)	68.90 (18.12)	63.29 (19.64)	<0.001
Attitude	26.81 (3.75)	27.11 (3.61)	23.70 (3.78)	<0.001
Skill	20.96 (4.47)	20.96 (4.45)	21.05 (4.70)	0.671
Mental health literacy	116.16 (20.74)	116.96 (20.45)	108.04 (21.89)	<0.001

**Table 2:** Characteristics of participants by anxiety symptom status.

	[Al l]	No Anxiety Symptoms	Anxiety Symptoms	*p*. Overall
	*N = 5759*	*N = 5013*	*N = 746*	
Age:				0.001
13_and_below	1532 (26.60%)	1368 (27.29%)	164 (21.98%)	
14_15	2137 (37.11%)	1874 (37.38%)	263 (35.25%)	
16_17	1843 (32.00%)	1563 (31.18%)	280 (37.53%)	
18_and_above	247 (4.29%)	208 (4.15%)	39 (5.23%)	
Gender:				<0.001
male	2703 (46.94%)	2436 (48.59%)	267 (35.79%)	
female	3056 (53.06%)	2577 (51.41%)	479 (64.21%)	
Residence:				0.008
Urban	4432 (76.96%)	3829 (76.38%)	603 (80.83%)	
Rural	1327 (23.04%)	1184 (23.62%)	143 (19.17%)	
Exercise:				<0.001
Never/almost never	532 (9.24%)	399 (7.96%)	133 (17.83%)	
1–3 times/month	1318 (22.89%)	1111 (22.16%)	207 (27.75%)	
1–2 times/week	1831 (31.79%)	1632 (32.56%)	199 (26.68%)	
3–5 times/week	910 (15.80%)	817 (16.30%)	93 (12.47%)	
Daily or almost daily	1168 (20.28%)	1054 (21.03%)	114 (15.28%)	
Eating habit:				<0.001
Regular three meals a day	4886 (84.84%)	4379 (87.35%)	507 (67.96%)	
Regular two meals a day	319 (5.54%)	246 (4.91%)	73 (9.79%)	
Regular multiple meals a day (more than three)	281 (4.88%)	226 (4.51%)	55 (7.37%)	
Irregular eating pattern	273 (4.74%)	162 (3.23%)	111 (14.88%)	
Spend on electronic screen devices:				<0.001
Less than 2 h	3860 (67.03%)	3477 (69.36%)	383 (51.34%)	
2 to less than 4 h	1156 (20.07%)	938 (18.71%)	218 (29.22%)	
4 to less than 6 h	458 (7.95%)	382 (7.62%)	76 (10.19%)	
6 to less than 8 h	164 (2.85%)	133 (2.65%)	31 (4.16%)	
8 h or more	121 (2.10%)	83 (1.66%)	38 (5.09%)	
Parental parenting style:				<0.001
Strict father, kind mother	1224 (21.25%)	1062 (21.18%)	162 (21.72%)	
Kind father, strict mother	979 (17.00%)	868 (17.31%)	111 (14.88%)	
Strict father, strict mother	609 (10.57%)	475 (9.48%)	134 (17.96%)	
Kind father, kind mother	2292 (39.80%)	2077 (41.43%)	215 (28.82%)	
Not applicable	655 (11.37%)	531 (10.59%)	124 (16.62%)	
Parental Relationship:				<0.001
Very harmonious	2309 (40.09%)	2148 (42.85%)	161 (21.58%)	
Relatively harmonious	2401 (41.69%)	2093 (41.75%)	308 (41.29%)	
Average	787 (13.67%)	605 (12.07%)	182 (24.40%)	
Not very harmonious	180 (3.13%)	113 (2.25%)	67 (8.98%)	
Very disharmonious	82 (1.42%)	54 (1.08%)	28 (3.75%)	
Knowledge	68.39 (18.33)	68.97 (18.06)	64.49 (19.59)	<0.001
Attitude	26.81 (3.75)	27.21 (3.58)	24.08 (3.73)	<0.001
Skill	20.96 (4.47)	20.92 (4.43)	21.25 (4.71)	0.070
Mental health literacy	116.16 (20.74)	117.11 (20.34)	109.82 (22.24)	<0.001

**Table 3:** The association between adolescent MHL and depression risk.

Categories	Model 1			Model 2			Model 3		
	OR (95% CI)	*p*-Value	P for Trend	OR (95% CI)	*p*-Value	P for Trend	OR (95% CI)	*p*-Value	P for Trend
Mental Health Literacy									
Continuous variable per unit	0.98 (0.98, 0.99)	<0.001		0.97 (0.97, 0.98)	<0.001		0.98 (0.98, 0.99)	<0.001	
Quartile			<0.001			<0.001			<0.001
Q1 (N = 1440)	Ref			Ref			Ref		
Q2 (N = 1440)	0.67 (0.53, 0.83)	<0.001		0.62 (0.49, 0.78)	<0.001		0.76 (0.59, 0.97)	0.030	
Q3 (N = 1440)	0.50 (0.39, 0.64)	<0.001		0.44 (0.34, 0.56)	<0.001		0.54 (0.41, 0.71)	<0.001	
Q4 (N = 1439)	0.29 (0.22, 0.39)	<0.001		0.25 (0.18, 0.33)	<0.001		0.32 (0.23, 0.43)	<0.001	
Knowledge									
Continuous variable per unit	0.98 (0.98, 0.99)	<0.001		0.98 (0.98, 0.98)	<0.001		0.98 (0.98, 0.99)	<0.001	
Quartile			<0.001			<0.001			<0.001
Q1 (N = 1440)	Ref			Ref			Ref		
Q2 (N = 1440)	0.65 (0.50, 0.83)	<0.001		0.58 (0.45, 0.75)	<0.001		0.67 (0.51, 0.87)	0.004	
Q3 (N = 1440)	0.67 (0.52, 0.85)	0.001		0.58 (0.45, 0.74)	<0.001		0.70 (0.53, 0.90)	0.007	
Q4 (N = 1439)	0.48 (0.37, 0.63)	<0.001		0.39 (0.30, 0.50)	<0.001		0.45 (0.34, 0.60)	<0.001	
Attitudes									
Continuous variable per unit	0.80 (0.78, 0.82)	<0.001		0.80 (0.78, 0.82)	<0.001		0.83 (0.81, 0.86)	<0.001	
Quartile			<0.001			<0.001			<0.001
Q1 (N = 1440)	Ref			Ref			Ref		
Q2 (N = 1440)	0.44 (0.35, 0.54)	<0.001		0.44 (0.36, 0.55)	<0.001		0.53 (0.42, 0.67)	<0.001	
Q3 (N = 1440)	0.14 (0.10, 0.19)	<0.001		0.14 (0.10, 0.20)	<0.001		0.19 (0.13, 0.27)	<0.001	
Q4 (N = 1439)	0.10 (0.07, 0.15)	<0.001		0.11 (0.07, 0.17)	<0.001		0.19 (0.12, 0.28)	<0.001	
Skills									
Continuous variable per unit	1.00 (0.98, 1.03)	0.66		1.01 (0.99, 1.03)	0.38		1.01 (0.99, 1.03)	0.34	
Quartile			0.539			0.277			0.360
Q1 (N = 1440)	Ref			Ref			Ref		
Q2 (N = 1440)	1.03 (0.80, 1.33)	0.804		1.03 (0.79, 1.33)	0.846		1.09 (0.83, 1.44)	0.523	
Q3 (N = 1440)	1.01 (0.79, 1.29)	0.952		1.02 (0.80, 1.31)	0.852		1.09 (0.84, 1.41)	0.527	
Q4 (N = 1439)	1.10 (0.85, 1.41)	0.475		1.16 (0.90, 1.50)	0.247		1.15 (0.87, 1.51)	0.327	

**Table 4:** The association between adolescent MHL and anxiety risk.

Categories	Model 1			Model 2			Model 3		
	OR (95% CI)	*p*-Value	*p* for Trend	OR (95% CI)	*p*-Value	*p* for Trend	OR (95% CI)	*p*-Value	*p* for Trend
Mental Health Literacy									
Continuous variable per unit	0.98 (0.98, 0.99)	<0.001		0.98 (0.98, 0.99)	<0.001		0.98 (0.98, 0.99)	<0.001	
Quartile			<0.001			<0.001			<0.001
Q1 (N = 1440)	Ref			Ref			Ref		
Q2 (N = 1440)	0.69 (0.57, 0.85)	<0.001		0.66 (0.54, 0.81)	<0.001		0.75 (0.60, 0.93)	0.008	
Q3 (N = 1440)	0.55 (0.45, 0.68)	<0.001		0.51 (0.41, 0.63)	<0.001		0.59 (0.47, 0.74)	<0.001	
Q4 (N = 1439)	0.39 (0.31, 0.49)	<0.001		0.34 (0.27, 0.44)	<0.001		0.41 (0.32, 0.53)	<0.001	
Knowledge									
Continuous variable per unit	0.99 (0.98, 0.99)	<0.001		0.98 (0.98, 0.99)	<0.001		0.99 (0.98, 0.99)	<0.001	
Quartile			<0.001			<0.001			<0.001
Q1 (N = 1440)	Ref			Ref			Ref		
Q2 (N = 1440)	0.75 (0.60, 0.92)	0.007		0.69 (0.56, 0.86)	<0.001		0.77 (0.61, 0.96)	0.022	
Q3 (N = 1440)	0.56 (0.45, 0.69)	<0.001		0.50 (0.40, 0.63)	<0.001		0.57 (0.45, 0.72)	<0.001	
Q4 (N = 1439)	0.65 (0.53, 0.80)	<0.001		0.56 (0.45, 0.69)	<0.001		0.64 (0.50, 0.80)	<0.001	
Attitudes									
Continuous variable per unit	0.80 (0.78, 0.82)	<0.001		0.80 (0.79, 0.82)	<0.001		0.84 (0.82, 0.86)	<0.001	
Quartile			<0.001			<0.001			<0.001
Q1 (N = 1440)	Ref			Ref			Ref		
Q2 (N = 1440)	0.45 (0.38, 0.54)	<0.001		0.45 (0.38, 0.55)	<0.001		0.53 (0.44, 0.65)	<0.001	
Q3 (N = 1440)	0.19 (0.15, 0.25)	<0.001		0.20 (0.16, 0.26)	<0.001		0.26 (0.20, 0.34)	<0.001	
Q4 (N = 1439)	0.10 (0.07, 0.14)	<0.001		0.11 (0.08, 0.16)	<0.001		0.17 (0.12, 0.24)	<0.001	
Skills									
Continuous variable per unit	1.02 (1.00, 1.03)	0.060		1.02 (1.00, 1.04)	0.027		1.02 (1.00, 1.04)	0.049	
Quartile			0.036			0.014			0.045
Q1 (N = 1440)	Ref			Ref			Ref		
Q2 (N = 1440)	1.04 (0.83, 1.29)	0.738		1.03 (0.83, 1.29)	0.766		1.08 (0.85, 1.36)	0.538	
Q3 (N = 1440)	1.03 (0.84, 1.28)	0.752		1.04 (0.84, 1.29)	0.703		1.07 (0.85, 1.34)	0.561	
Q4 (N = 1439)	1.27 (1.03, 1.57)	0.025		1.33 (1.07, 1.64)	0.010		1.29 (1.02, 1.62)	0.031	

## Data Availability

The datasets generated and analyzed during the current study are not publicly available due to institutional restrictions on participant confidentiality but may be made available from the corresponding author upon reasonable request.

## Supplementary Materials

The following supporting information can be downloaded at https://www.mdpi.com/article/10.3390/bs16061027/s1, Figure S1: Test set calibration plot. A: Test set calibration plot of depression. B: Test set calibration plot of anxiety; Table S1: Characteristics of participants by depressive symptom status; Table S2: Characteristics of participants by anxiety symptom status; Table S3: Other performance metrics regarding machine learning of depression; Table S4: Other performance metrics regarding machine learning of anxiety.

## Abbreviations

MHL	Mental Health Literacy
MHLQ	Mental Health Literacy Questionnaire
CES-D	Center for Epidemiologic Studies Depression Scale
GAD-7	Generalized Anxiety Disorder Scale-7
OC	Only Child
LOC	Living on Campus
FES	Family Economic Situation
EH	Eating Habit
SOESD	Spend on Electronic Screen Devices
CMS	Current Myopia Status
BMI	Body Mass Index
PPS	Parental Parenting Style
PR	Parental Relationship
ML	Machine Learning
RCS	Restricted Cubic Spline
DT	Decision Tree
RF	Random Forest
LR	Logistic Regression
GBDT	Gradient Boosting Decision Tree
KNNC	K-Nearest Neighbor Classification
ET	Extra Tree
XGBoost	eXtreme Gradient Boosting
NB	Naive Bayes
AdaBoost	Adaptive Boosting
LightGBM	Light Gradient Boosting Machine
SHAP	Shapley Additive Interpretation
AUC	Receiver Operating Characteristic Curve
PPV	Positive Predictive Value
NPV	Negative Predictive Value
