# Supplementary material for: Association Between Mental Health Literacy and Its Dimensions with Adolescent Depression and Anxiety: A Cross-Sectional Study Among 5759 Adolescents in China
Source: Behav Sci (Basel). 2026 Jun 18;16(6):1027. doi: 10.3390/bs16061027 (PMC13295392; doi:10.3390/bs16061027)

Supplementary material

Table-S1. Characteristics of participants by depressive symptom status.

	All<br>*N=5759*	No depressive symptoms<br>*N=5243*	Depressive symptoms<br>*N=516*	p. overall
Grade:				<0.001
Grade7	1153 (20.02%)	1095 (20.88%)	58 (11.24%)	
Grade8	995 (17.28%)	926 (17.66%)	69 (13.37%)	
Grade9	906 (15.73%)	813 (15.51%)	93 (18.02%)	
Grade10	967 (16.79%)	861 (16.42%)	106 (20.54%)	
Grade11	859 (14.92%)	772 (14.72%)	87 (16.86%)	
Grade12	879 (15.26%)	776 (14.80%)	103 (19.96%)	
Only child:				0.658
Yes	1223 (21.24%)	1109 (21.15%)	114 (22.09%)	
No	4536 (78.76%)	4134 (78.85%)	402 (77.91%)	
Boarding:				0.914
Yes	2954 (51.29%)	2691 (51.33%)	263 (50.97%)	
No	2805 (48.71%)	2552 (48.67%)	253 (49.03%)	
Family economic situation:				<0.001
affluent	96 (1.67%)	89 (1.70%)	7 (1.36%)	
above average	811 (14.08%)	744 (14.19%)	67 (12.98%)	
average	3768 (65.43%)	3469 (66.16%)	299 (57.95%)	
below average	891 (15.47%)	772 (14.72%)	119 (23.06%)	
difficult	193 (3.35%)	169 (3.22%)	24 (4.65%)	
Current myopia status:				0.026
No myopia	1577 (27.38%)	1462 (27.88%)	115 (22.29%)	
Myopia less than 100 degrees	746 (12.95%)	681 (12.99%)	65 (12.60%)	
Myopia 100-299 degrees	1789 (31.06%)	1621 (30.92%)	168 (32.56%)	
Myopia 300-599 degrees	1428 (24.80%)	1288 (24.57%)	140 (27.13%)	
Myopia 600 degrees or more	219 (3.80%)	191 (3.64%)	28 (5.43%)	
BMI:				0.260
Underweight	2217 (38.50%)	2029 (38.70%)	188 (36.43%)	
Normal weight	2737 (47.53%)	2487 (47.43%)	250 (48.45%)	
Overweight	447 (7.76%)	397 (7.57%)	50 (9.69%)	
Obese	358 (6.22%)	330 (6.29%)	28 (5.43%)	

Table-S2. Characteristics of participants by anxiety symptom status.

	**Al l** *N=5759*	**No anxiety symptoms** *N=5013*	**Anxiety symptoms** *N=746*	**p. overall**
Grade:				<0.001
Grade7	1153 (20.02%)	1037 (20.69%)	116 (15.55%)	
Grade8	995 (17.28%)	896 (17.87%)	99 (13.27%)	
Grade9	906 (15.73%)	789 (15.74%)	117 (15.68%)	
Grade10	967 (16.79%)	832 (16.60%)	135 (18.10%)	
Grade11	859 (14.92%)	728 (14.52%)	131 (17.56%)	
Grade12	879 (15.26%)	731 (14.58%)	148 (19.84%)	
Only child:				0.384
Yes	1223 (21.24%)	1055 (21.05%)	168 (22.52%)	
No	4536 (78.76%)	3958 (78.95%)	578 (77.48%)	
Boarding:				1.000
Yes	2954 (51.29%)	2571 (51.29%)	383 (51.34%)	
No	2805 (48.71%)	2442 (48.71%)	363 (48.66%)	
Family economic situation:				<0.001
affluent	96 (1.67%)	85 (1.70%)	11 (1.47%)	
above average	811 (14.08%)	715 (14.26%)	96 (12.87%)	
average	3768 (65.43%)	3316 (66.15%)	452 (60.59%)	
below average	891 (15.47%)	738 (14.72%)	153 (20.51%)	
difficult	193 (3.35%)	159 (3.17%)	34 (4.56%)	
Current myopia status:				<0.001
No myopia	1577 (27.38%)	1408 (28.09%)	169 (22.65%)	
Myopia less than 100 degrees	746 (12.95%)	656 (13.09%)	90 (12.06%)	
Myopia 100-299 degrees	1789 (31.06%)	1546 (30.84%)	243 (32.57%)	
Myopia 300-599 degrees	1428 (24.80%)	1230 (24.54%)	198 (26.54%)	
Myopia 600 degrees or more	219 (3.80%)	173 (3.45%)	46 (6.17%)	
BMI:				0.186
Underweight	2217 (38.50%)	1927 (38.44%)	290 (38.87%)	
Normal weight	2737 (47.53%)	2389 (47.66%)	348 (46.65%)	
Overweight	447 (7.76%)	377 (7.52%)	70 (9.38%)	
Obese	358 (6.22%)	320 (6.38%)	38 (5.09%)	

Table S3. Other performance metrics regarding machine learning of depression.

Model Name	Accuracy	Sensitivity	Specificity	PPV	NPV	F1	AUC
**Training set (N = 4031)**							
KNN	0.8799	0.5079	0.9163	0.3747	0.95	0.4319	0.8294
Extra Trees	0.8896	0.4598	0.9319	0.399	0.9461	0.4273	0.8239
Gradient Boosting	0.8779	0.4349	0.9215	0.3528	0.9431	0.3896	0.8163
LightGBM	0.8854	0.4017	0.933	0.3708	0.9407	0.3856	0.8159
XGBoost	0.8653	0.4737	0.9038	0.3263	0.9458	0.3864	0.8106
Random Forest	0.8807	0.4294	0.9251	0.3605	0.9428	0.3919	0.8075
AdaBoost	0.8445	0.5125	0.8771	0.2909	0.9428	0.3711	0.7986
Logistic Regression	0.8606	0.4626	0.8997	0.3121	0.9445	0.3728	0.7921
Naive Bayes	0.8378	0.5208	0.8689	0.281	0.9485	0.365	0.7863
Decision Tree	0.8685	0.4127	0.9134	0.3191	0.9405	0.3599	0.7853
**Test set (N = 1728)**							
Extra Trees	0.8802	0.3742	0.9301	0.3452	0.9378	0.3591	0.7937
Logistic Regression	0.8611	0.4387	0.9027	0.3077	0.9423	0.3617	0.7902
Naive Bayes	0.8322	0.4839	0.8665	0.2632	0.9446	0.3409	0.7886
Gradient Boosting	0.8831	0.3677	0.9339	0.354	0.9375	0.3608	0.7848
Random Forest	0.8733	0.3484	0.925	0.314	0.9351	0.3303	0.7846
XGBoost	0.8605	0.3871	0.9072	0.2913	0.9376	0.3324	0.7835
LightGBM	0.8872	0.3226	0.9428	0.3571	0.9339	0.339	0.7797
AdaBoost	0.8414	0.4774	0.8773	0.2772	0.9446	0.3607	0.7791
KNN	0.8646	0.4	0.9104	0.3054	0.939	0.3464	0.7713
Decision Tree	0.8663	0.3161	0.9205	0.2816	0.9315	0.2979	0.7496

Table S4. Other performance metrics regarding machine learning of anxiety.

Model Name	Accuracy	Sensitivity	Specificity	PPV	NPV	F1	AUC
**Training set (N = 4031)**							
Random Forest	0.8432	0.3563	0.9156	0.3859	0.9053	0.3705	0.777
Gradient Boosting	0.8574	0.3295	0.9359	0.4332	0.9037	0.3743	0.7769
LightGBM	0.84	0.3774	0.9088	0.381	0.9075	0.3792	0.7767
XGBoost	0.8385	0.3774	0.9071	0.3767	0.9074	0.377	0.7767
Extra Trees	0.8611	0.3276	0.9404	0.45	0.9039	0.3792	0.7762
Decision Tree	0.8167	0.4751	0.8675	0.3478	0.9174	0.4016	0.7712
AdaBoost	0.839	0.3678	0.9091	0.3757	0.9062	0.3717	0.7693
Logistic Regression	0.8323	0.3774	0.9	0.3595	0.9067	0.3682	0.7635
KNN	0.8623	0.3429	0.9396	0.4578	0.9058	0.3921	0.759
Naive Bayes	0.8142	0.3966	0.8763	0.3229	0.9071	0.356	0.7584
**Test set (N = 1728)**							
Logistic Regression	0.8345	0.3705	0.9036	0.364	0.906	0.3673	0.7775
XGBoost	0.8333	0.3571	0.9043	0.3571	0.9043	0.3571	0.777
LightGBM	0.8339	0.3438	0.9069	0.3548	0.9027	0.3492	0.7762
Gradient Boosting	0.8519	0.2991	0.9342	0.4036	0.8995	0.3436	0.7753
Random Forest	0.8391	0.3348	0.9142	0.3676	0.9022	0.3505	0.7752
AdaBoost	0.8368	0.3438	0.9102	0.3632	0.903	0.3532	0.7733
Extra Trees	0.8547	0.2857	0.9395	0.4129	0.8983	0.3377	0.7727
Naive Bayes	0.8154	0.3884	0.879	0.3234	0.9061	0.3529	0.7632
Decision Tree	0.8125	0.4375	0.8684	0.3311	0.912	0.3769	0.757
KNN	0.8513	0.3036	0.9328	0.4024	0.8999	0.3461	0.7174

Figure S1. Test set calibration plot. A: Test set calibration plot of depression. B: Test set calibration plot of anxiety.

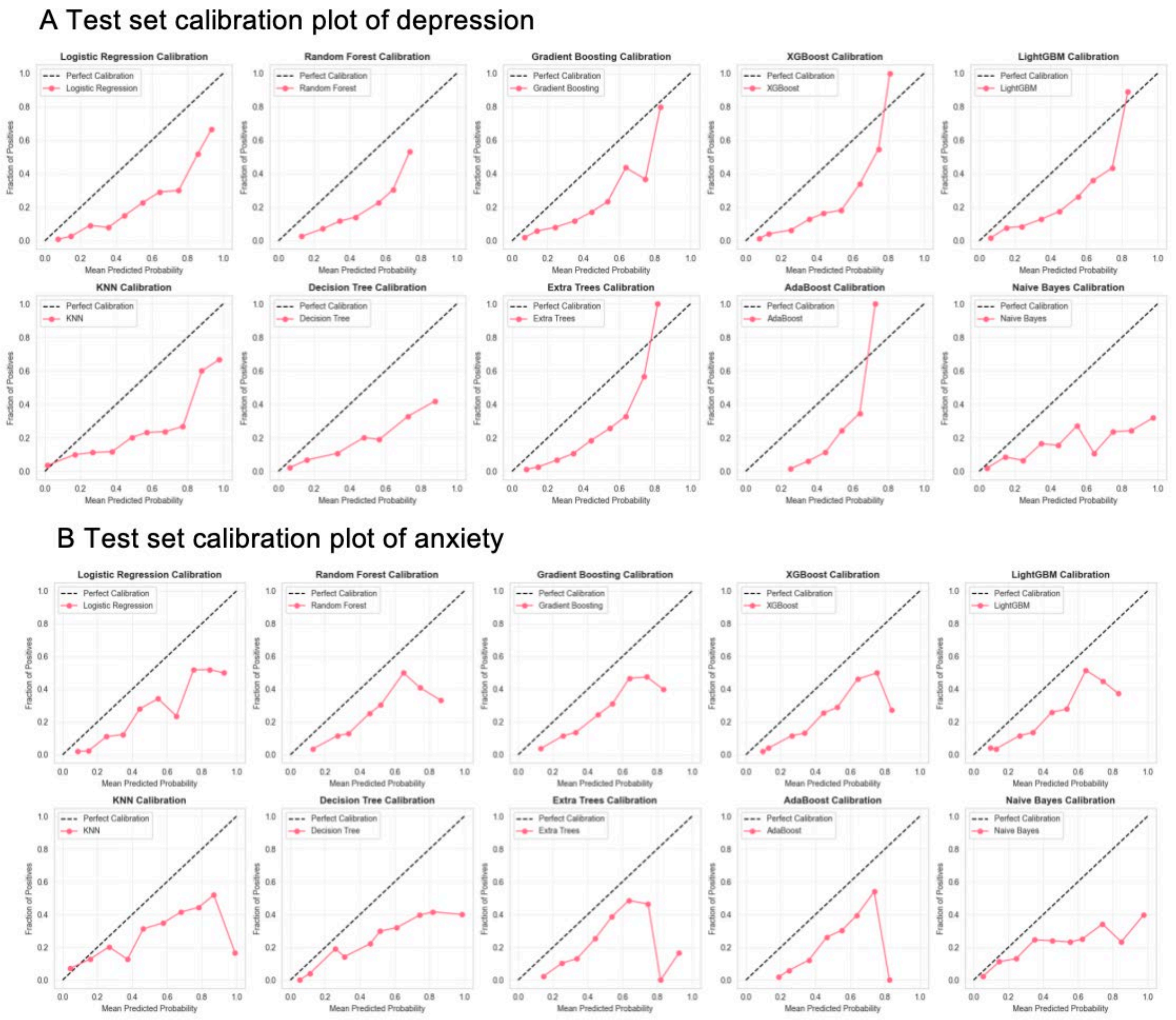

Supplement: Supplementary file 1 [file behavsci-16-01027-s001.zip › behavsci-4225091-supplementary.pdf]
